# Dysregulations of sonic hedgehog signaling in *MED12*‐related X‐linked intellectual disability disorders

**DOI:** 10.1002/mgg3.569

**Published:** 2019-02-06

**Authors:** Siddharth Srivastava, Tejasvi Niranjan, Melanie M. May, Patrick Tarpey, William Allen, Anna Hackett, Pierre‐Simon Jouk, Lucy Raymond, Slyvain Briault, Cindy Skinner, Annick Toutain, Jozef Gecz, William Heath, Roger E. Stevenson, Charles E. Schwartz, Tao Wang

**Affiliations:** ^1^ Institute of Genetic Medicine and Department of Pediatrics Johns Hopkins University Baltimore Maryland; ^2^ Greenwood Genetic Center Greenwood South Carolina; ^3^ Welcome Trust Sanger Institute Cambridge UK; ^4^ Fullerton Genetics Asheville North Carolina; ^5^ Genetics of Learning Disability Service Hunter Genetics Waratah NSW Australia; ^6^ Service de Génétique Clinique Hôpital Couple‐Enfant Grenoble France; ^7^ Cambridge Institute of Medical Research Cambridge UK; ^8^ Centre Hospitalier Régional d’Orléans Orléans France; ^9^ Service de Génétique Clinique, Hôpital Bretonneau Tours France; ^10^ Adelaide Medical School and Robinson Research Institute The University of Adelaide Adelaide Australia; ^11^ J.I. Riddle Developmental Center Morganton North Carolina

**Keywords:** *MED12*, Mutation, qRT‐PCR, SHH Signaling, XLID

## Abstract

**Background:**

Mutations in mediator of RNA polymerase II transcription subunit 12 homolog (*MED12*, OMIM 300188) cause X‐linked intellectual disability (XLID) disorders including FG, Lujan, and Ohdo syndromes. The Gli3‐dependent Sonic Hedgehog (SHH) signaling pathway has been implicated in the original FG syndrome and Lujan syndrome. How are SHH‐signaling defects related to the complex clinical phenotype of *MED12*‐associated XLID syndromes are not fully understood.

**Methods:**

Quantitative RT‐PCR was used to study expression levels of three SHH‐signaling genes in lymophoblast cell lines carrying four *MED12* mutations from four unrelated XLID families. Genotype and phenotype correlation studies were performed on these mutations.

**Results:**

Three newly identified and one novel *MED12* mutations in six affected males from four unrelated XLID families were studied. Three mutations (c.2692A>G; p.N898D, c.3640C>T; p.R1214C, and c.3884G>A; p.R1295H) are located in the LS domain and one (c.617G>A; p.R206Q) is in the L domain of MED12. These mutations involve highly conserved amino acid residues and segregate with ID and related congenital malformations in respective probands families. Patients with the LS‐domain mutations share many features of FG syndrome and some features of Lujan syndrome. The patient with the L‐domain mutation presented with ID and predominant neuropsychiatric features but little dysmorphic features of either FG or Lujan syndrome. Transcript levels of three Gli3‐dependent SHH‐signaling genes, *CREB5*, *BMP4*, and *NEUROG2*, were determined by quantitative RT‐PCR and found to be significantly elevated in lymphoblasts from patients with three mutations in the MED12‐LS domain.

**Conclusions:**

These results support a critical role of MED12 in regulating Gli3‐dependent SHH signaling and in developing ID and related congenital malformations in XLID syndromes. Differences in the expression profile of SHH‐signaling genes potentially contribute to variability in clinical phenotypes in patients with *MED12*‐related XLID disorders.

## 1 INTRODUCTION

Mediator of RNA polymerase II transcription subunit 12 homolog (*MED12*) encodes a core component of a multi‐subunit mediator complex, which includes MED12, MED13, cyclin C, and CDK8 (Yin & Wang, 2014). Mediator is a large, evolutionarily conserved protein complex that plays an important role in gene regulation (Yin & Wang, 2014). The mediator complex consists of approximately 30 subunits that are assembled into four functional modules interacting with signaling proteins in the vertebral endoderm (Shin et al., 2008) and neuronal development (Wang, Yang, Uno, Roeder, & Guo, 2006) and the canonical Wnt signaling pathway (Rocha, Scholze, Bleiss, & Schrewe, 2010). GLI family zinc family protein 3 (GLI3) binds directly to the PQL domain of MED12, which suppresses the enhanced GLI3‐dependent transactivation induced by Sonic hedgehog (SHH) signaling (Zhou, Kim, Ishii, & Boyer, 2006). SHH signaling has critical functions in the development of the central nervous system including neural tube patterning, neuronal cell differentiation and survival (Ho & Scott, 2002).

*MED12* mutations have been associated with a wide phenotypic spectrum of *MED12*‐related disorders (Charzewska et al., 2018). Somatic mutations in *MED12* have been reported in leiomyosarcoma (Ravegnini et al., 2013) and prostate cancer (Kämpjärvi et al., 2015). Germline *MED12* mutations were originally found in patients with FG and Lujan syndromes (Risheg et al., 2007; Schwartz et al., 2007) and later in patients with X‐linked Ohdo syndrome and isolated XLID disorders (Bouazzi, Lesca, Trujillo, Alwasiyah, & Munnich, 2015; Callier et al., 2013; Isidor et al., 2013; Langley et al., 2015; Lesca et al., 2013; Tzschach et al., 2015; Vulto‐van Silfhout et al., 2013; Yamamoto & Shimojima, 2015). Mutations that cause FG syndrome (p.R961W) and Lujan syndrome (p.N1007S) were found to compromise the mediator‐imposed constraint on GLI3‐dependent SHH signaling and result in increased transcript levels for multiple GLI3 target genes in lymphoblasts from patients (Zhou et al., 2012). How GLI3–SHH‐signaling defects relate to the complex clinical phenotypes of *MED12*‐associated XLID syndromes are not known.

We report genetic and functional characterizations of four *MED12* mutations in patients with XLID disorders. Patients with LS‐domain mutations show ID and congenital malformations overlapping with FG and Lujan syndromes. Transcript levels of three SHH/GLI3‐signaling genes, *BMP4*(OMIM112262), *CREB5*, *NEUROG2* (OMIM606624), were found to be significantly elevated in lymphoblasts from patients with these mutations. The patient with a change in the L domain only had elevated expression of *BMP4* and presented with ID and predominant psychiatric phenotypes. These results expand genotype and phenotype spectrums of *MED12*‐mediated XLID syndromes and support a critical role of MED12‐regulated Gli3‐dependent SHH signaling in the expression of disease phenotypes.

## 2 MATERIALS AND METHODS

### 2.1 Study patients and controls

Patients with XLID and control males with normal cognitive function were recruited by the Greenwood Genetic Center (Greenwood, SC) and the Johns Hopkins University (Baltimore, MD). Human subject research protocols for these studies were approved by Institutional Review Boards (IRBs) at the respective institutions. An informed consent was obtained from each study patient and/or their parents or legal guardians. These patients were all evaluated by clinical geneticists and underwent comprehensive laboratory studies for ID. All patients were found to have a normal karyotype, negative molecular testing for fragile X syndrome, and a negative screen for common inborn errors of metabolism. For each individual, 5–10 ml of blood was collected to establish EB‐transformed lymphoblast cell lines. Genomic DNA samples from affected probands males with XLID were used for sequencing and mutation screening. A cohort of >800 males with normal cognitive function from the Greenwood Genetic Center and Johns Hopkins University were used as controls. Additional reference data include the male portion of samples (*n* = 525) from the 1,000 Genomes project (Integrated Phase 1, version 3:20101123).

### 2.2 X chromosome exome sequencing and exon‐based resequencing

Sequence libraries were prepared using a TruSeq^TM^ Genomic DNA Library Preparation kit (Illumina), enriched for the human X chromosome Exome using a SureSelect Target Enrichment kit (Agilent), and sequenced using the 75 bp pair‐end sequence module on the HiSeq2000 (Illumina). Alignment of the fastq reads, base recalibration, and variant calling were completed using Bowtie2 and Unified Genotyper (GATK). Disease‐causing mutations were enriched using the nonclinical portion of the dbSNP, the male‐restricted portion of the 1,000 Genomes, Exome Variant Server datasets, and an affected sib‐pair/cross‐cohort‐based algorithm (Niranjan et al., 2015). Evolutionary conservation of the amino acid residues involved in the identified mutations was evaluated by multiple sequence alignment of HomoloGene (http://www.ncbi.nlm.nih.gov/homologene/). Standard bioinformatics algorithms including SIFT (http://sift.jcvi.org) and PolyPhen‐2 (http://genetics.bwh.harvard.edu/pph2/index.shtml) were used to predict the functional impact of the identified mutations (Niranjan et al., 2015). Additional exon‐based resequencing was conducted to identify novel *MED12* mutations in patients with intellectual disability and a pedigree consistent with X‐linked inheritance (Raymond et al., 2007). Sanger sequencing was used for validation, segregation analysis, and polymorphism studies of each mutation using the BigDye Terminator v3.1 Cycle Sequencing Kit on an ABI3100 automatic DNA analyzer (Applied Biosystems) following manufacturer's instruction. Variant analysis of *MED12* (NM_005120.2) was completed using standard sequence alignment software (CodonCode and MacVector) followed by manual investigations of the chromatograms.

### 2.3 Real‐time quantitative PCR analysis for transcript levels of SHH‐signaling genes

EBV‐transformed lymphoblastoid cell lines from patients and controls were cultured in RPMI 1640 medium with 15% FBS (Sigma) and 1% penicillin–streptomycin (100 U penicillin; 0.1 mg/ml streptomycin) at 37°C, 10% CO_2_. Cells were harvested at log phase. Total RNA was prepared from cultured lymphoblasts from individual patients and normal controls using a Qiagen RNA preparation kit. cDNA from these samples was synthesized using a M‐MLV RT kit (Promega). Real‐time qPCR was conducted using an absolute SYBR Green mix (Applied Biosystems) in an iCycler (BioRad). After initial denaturation at 95.0ºC for 3 min, the reaction was cycled for 35 times at 95.0ºC for 30 s and 60.0ºC for 45 s. Input samples were normalized using a simultaneous quantification of β‐actin. qPCR Primers used in this study are given in Table S1. Lymphoblast cell lines from three unrelated normal males and from the original Lujan family with a p.N1007S mutation were used as controls. Each data set for the transcript levels was generated from triplicate studies. Statistical analyses of qPCR data were performed using two‐tailed *t* test for comparison of the means of two independent samples. Data were presented as mean ± *SEM*; *p* < 0.05 was considered statistically significant.

## 3 RESULTS

### 3.1 Identification and pedigree analysis of *MED12* mutations in XLID families

**Figure 1:**
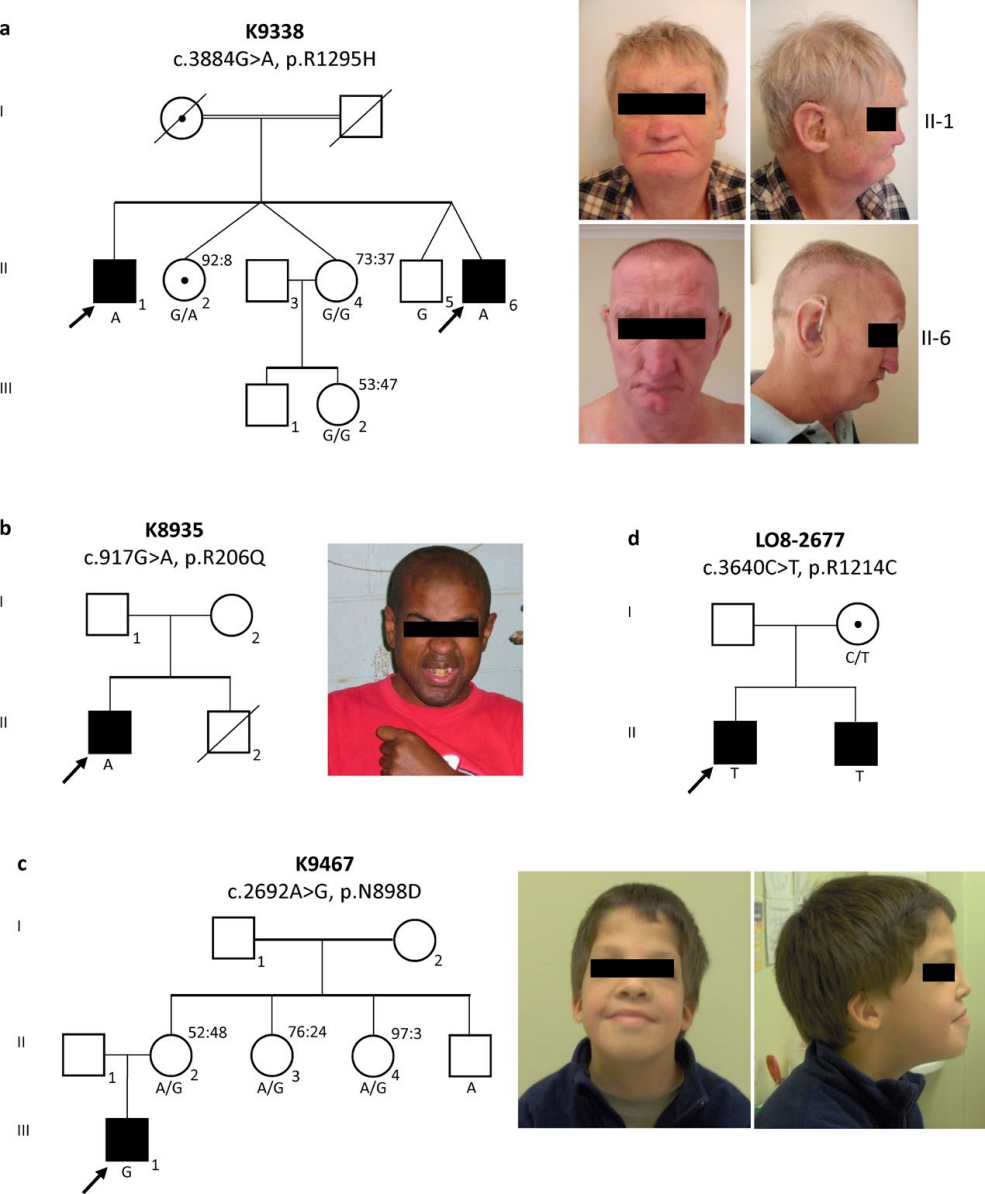
Pedigree Analysis and Clinical Features of XLID Probands with *MED12* Mutations. Panel a: Segregation of c.3884G>A; p.R1295H in pedigree of K9338; confirmed genotypes (WT, G; mutation, A) are indicated below individual symbols; skewed X chromosome inactivation data in female carriers are provided for II2, II4, III2. Facial features of one affected male are shown. Panel b: Segregation of c.617G>A; p.R206Q in pedigree K8935. Confirmed genotypes (WT, G; mutation, A) were shown below the affected male. Facial features of the affected male are shown. Panel c: Segregation of c.2692A>G; p.N898D in pedigree K9467. Confirmed genotypes (WT, A; mutation, G) are indicated below individual symbols; data for skewed X chromosome inactivation are provided for II2, II3, and II4. Facial features of the affected male are shown. Panel d: Segregation of c.3640C>T; p.R1214C in pedigree of LO8–2677. Confirmed genotypes (WT, C; mutation, T) are indicated below individual symbols. For all pedigrees: square symbol, male; circle symbol, female; open symbol, unaffected; filled symbol, affected; circle with a center dot, confirmed female carrier

**Figure 2:**
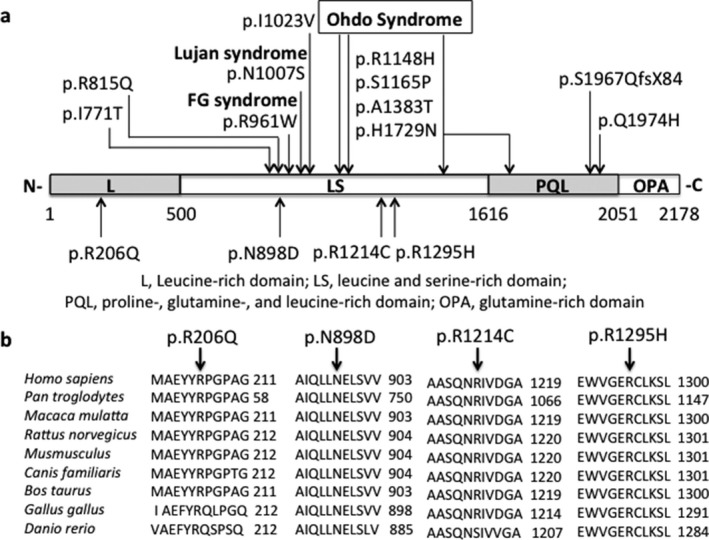
Disease‐causing *MED12* Mutations Involve Highly Conserved Amino Acid Residues and are Clustered around the Established Functional Domains. Panel a: Distribution of ID‐associated mutations over functional domains of MED12. Known ID‐associated mutations(Graham & Schwartz, 2013) are presented above and the four mutations of *MED12* (NM_005120.2) in this study are below the symbols of protein domains. Note that 3 mutations p.N898D, p.R1214C, and p.R1295H are located within the LS domain where the recurrent mutations for FG and Lujan syndromes occur and one, p.R206Q, is located within the L domain. L, leucine‐rich domain; LS, leucine serine‐rich domain; PQL, proline‐, glutamine‐, and leucine‐rich domain; OPA, glutamine‐rich domain. Panel b. *MED12*mutations involve highly evolutionarily conserved amino acid residues as shown in a multispecies sequence alignment. Mutations responsible for FG, Lujan, and Ohdo syndrome are based on previously published studies(Graham & Schwartz, 2013)

Exon resequencing of 718 X‐linked genes (Tarpey et al., 2009) found a c.3884G>A; p.R1295H mutation in two affected brothers (II‐3 and II‐6) and their mother who is heterozygous (K9338) (Figure 1a). Sequencing of *MED12* in a cohort of XLID families identified c.617G>A; p.R206Q mutation in an affected male and his unaffected mother (K8935) (Figure 1b) and a c.2692A>G; p.N898D mutation in an affected male and his unaffected mother in a family suspected to have FG syndrome (K9467) (Figure 1c). X chromosome exome sequencing identified a c.3640C>T; p.R1214C in two affected brothers and their unaffected mother in an XLID family (L08–2677) (Niranjan et al., 2015) (Figure 1d). The p.N898D, p.R1214C, and p.R1295H mutations are located in the LS domain while the p.R206Q mutation is located in the L domain of MED12 (Figure 2a). All four mutations involve highly conserved amino acid residues during evolution, and none was found in >1,325 normal X chromosomes (Figure 2b). The Combined Annotation Dependent Depletion (CADD) scores for all four mutations are in the range of 23.2 to 31 suggesting a high likelihood of deleterious effects (Table 1) (GRCh37‐v1.4, https://cadd.gs.washington.edu).

**Table 1:** Clinical features of MED12 mutations responsible for XLID syndromes

Pedigree ID_Syndrome	FG syndrome	Lujan syndrome	XLID_K8935_II‐1	XLID_K9467_III‐1	XLID_L08‐2677_II‐1	XLID_L08‐2677_II‐2	XLID_K9338_II‐1	XLID_K9338_II‐6
GRCh37‐v1.4			ChrX:70340884	ChrX:70346825	ChrX:70349228	ChrX:70349228	ChrX:70349901	ChrX:70349901
Mutation			c.617G>A; p.R206Q	c.2692A>G; p.N898D	c.3640C>T; p.R1214C	c.3640C>T; p.R1214C	c.3884G>A; p.R1295H	c.3884G>A; p.R1295H
CADD^a^			23.2	26.2	31	31	23.4	23.4
Evaluation Age (year)					15	17	14	11
Weight (kg)					5th centile	5th%	failure to thrive	failure to thrive
Height (cm)					10 th centile	5th%	25th centile (at 54 years)	60th centile (at 49 years)
OFC (cm)	Relative macrocephaly	Macrocephaly	Macrocephaly	>97th centile	50–75th centile	10–25th centile	Macrocephalic	75th centile (at 49 years)
Appearance		Marfanoid habitus	Height and weight >97%tile		Asthenic build	Asthenic build	Asthenic build	Poor muscle bulk
Motor Development				Delayed walking	Delayed walking	Delayed walking	Delayed; clumsy walking	Delayed; walking after 2 years
Language Development				Delayed; first word at 3 years	Delayed speech	Delayed speech	Speech unintellligible until 5 years	Delayed speech
Intellectual Disability	Moderate to severe	Mild to moderate	Mild to moderate (IQ 40–85)	Mild to moderate (IQ 58)	Mild to moderate	Mild to moderate	Mild to moderate (IQ 58)	Moderate
Behaviors	Friendly personality Short attention span Temper tantum	Hyperactivity Emotional lability Aggressiveness	Aggression Panic disorders Agoraphobia	Psychological lability Short attention span Easily frustrated	Friendly personality Excessive talkativeness	Friendly personality Excessive talkativeness	Impulsiveness Restlessness Temper tantrums	
Craniofacial	Tall forehead Frontal hair upsweep	Tall forehead		Tall and broad forehead Frontal upsweep	Tall forehead	Tall forehead		
	Long and narrow face Maxillary hypoplasia	Long and narrow face Maxillary hypoplasia		Maxillary hypoplasia	Long and narrow face Maxillary hypoplasia	Long and narrow face	Long face; prominent supraorbital ridge	Long face with maxillary hypoplasia
	Hypertelorism; downslanting palpebral fissures	Downslanting palpebral fissures	Downslanting palpebral fissures	Downslanting palpebral fissures Hypertelorism Astigmatism, hyeropia	Telecanthus	Telecanthus	Epicanthus; downslanting palpebral fissures; cataract	Strabismus
	Small prominent ears with simplified helical pattern	Hypernasal speech	Astigmatism, exotropia, and mild hyperopia	Small posteriorly rotated ears	Normal ears	Normal ears	Normal ears	
	Dental crowding Micro/retrognathia	Dental crowding; micro/retrognathia		Dental crowding and prognathism			High‐arched palate	High‐arched palate
Musculoskeletal	Pectus extracavatum Scoliosis Joint contracture				Mild pectus carinatum	Severe pectus carinatum Moderate scoliosis Mild contracture of elbows	Thoracic kyphosis;	Marked thoracic kyphosis
	Broad thumb; syndactyly; persistent fetal finger pads	Long extensible digits; broad thumbs	Long hands (length >97th%)	Angulation of distal phalanges; broad big toes	Hand length: 25–50th centile Middle finger length: 50th centile Arm span‐to‐height ratio: 1.03 Upper‐to‐lower segment ratio: 0.89	Hand length: 25–50th centile Middle finger length: 25–50th centile Arm span‐to‐height ratio: 1.05 Upper‐to‐lower segment ratio: 0.71 Slender fingers; hyperextensibility; fifth finger clinodactyly; pes planus	Pes cavus	Finger contractures at PIP joints; Hammer toes; wide gap between first and second toes
Gastrointestinal	Anal anomaly; constipation			Imperforate anus Constipation	Severe constipation; megacolon		Anal stricture and stenosis; umblical and inguinal hernia	Constipation
Genitourinary	Genitourinary anomaly		Hydroceles	Cryptorchidism		Posteriour urethral valves	Undescended testicles	Undescended testicles
Neurological	Hypotonia	Hypotonia		Hypotonia	Seizure disorders; wide spaced gait	Seizure disorders	Uncoordinated gait; hearing loss	Hypotonia; hearing loss
	Agenesis of corpus callosum	Agenesis of corpus callosum			Normal head CT	Normal head CT	Agenesis of corpus callosum; enlarged ventricules on MRI	

a Combined Annotation Dependent Depletion (CADD); https://cadd.gs.washington.edu

### 3.2 Increased expression of SHH‐signaling genes in lymphoblasts with LS‐domain mutations

**Figure 3:**
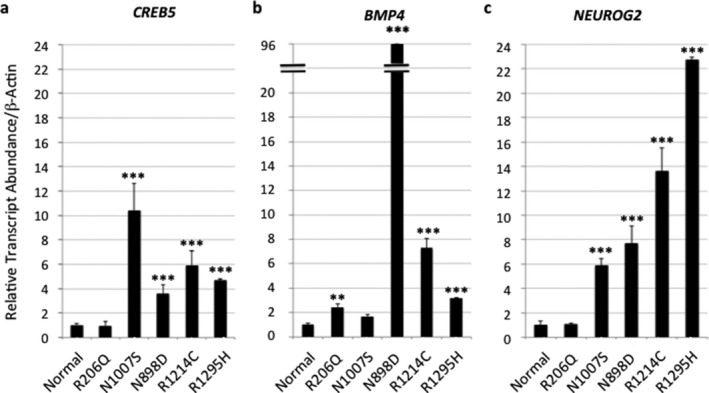
Real‐time Quantitative PCR Analysis of Transcript Levels of Three SHH‐Signaling Genes in Lymphoblasts from Patients with XLID. Note that compared to normal control lymphoblasts (*n* = 3), the transcript levels for all three genes, *CREB5, BMP4, NEUROG2* in the SHH‐signaling pathway show a significant increase in lymphoblasts carrying three *MED12* mutations (p.N898D; p.R1214C; and p.R1295H) within the LS domain but minimal changes for the mutation, p.R206Q, within the L domain. N1007S is the lymphoblast cell line from the probands of the original Lujan syndrome family(Schwartz et al., 2007). Means ± *SEM* from the triplicate studies of each lymphoblast cell line were shown. **, *p* < 0.01; ***, *p* < 0.001

Increased transcript levels for SHH‐signaling genes have been reported in cell lines from patients with FG and Lujan syndrome (Zhou et al., 2012). We thus performed a real‐time qRT‐PCR quantification of transcript levels of three genes (*CREB5*, *BMP4*, and *NEUROG2)*in the SHH‐signaling pathway in lymphoblasts from patients with four *MED12* mutations (Figure 3). Transcript levels for these genes are significantly increased for the three LS‐domain mutations. The L‐domain mutation, p.R206Q, shows only modest increase in the transcript of *BMP4* (Figure 3). Expression of *HPRT1*, a house‐keeping gene not known to be regulated by SHH/GLI3 signaling, showed no significant difference in lymphoblast cell lines between normal controls and patients with these *MED12* mutations (Figure S1). These results support that the MED12‐LS domain plays a major role in the negative regulation of Gli3‐dependent SHH signaling (Zhou et al., 2012), and these mutations found in patients with ID and congenital malformations show deleterious effects on MED12 function.

### 3.3 Expanding phenotypic spectrum of *MED12*‐related XLID disorders

*MED12*‐related XLID disorders exhibit a wide phenotypic spectrum in impairment of cognition, behavioral defects, and multiple congenital anomalies (Graham & Schwartz, 2013). “The patient with p.N898D mutation presented with macrocephaly, tall and broad forehead, downslanting palpebral fissures, small and posteriorly rotated ears, broad big toes, imperforate anus and constipation, and behavioral profile that mostly resemble patients with FG syndrome. Patients with p.R1214C and p.R1295H mutations presented with features that resemble both Lujan and FG syndromes. These features include craniofacial features, behavioral profile, anal anomalies, and severe constipation that are common in patients with FG syndrome as well as long and thin face, high‐arch palate, asthenic body build, reduced muscle mass, long and slender fingers, joint hyperextensibility that are seen in patients with Lujan syndrome. The patient with p.R206Q mutation presented with ID and predominant neuropsychiatric phenotype but without significant dysmorphic features or congenital malformations of either FG or Lujan syndromes (Table 1).”

## 4 DISCUSSION

Classical features of FG syndrome include ID, craniofacial features such as macrocephaly, prominent forehead, hypertelorism, downslanting palpebral fissures, small ears, musculoskeletal anomalies such as pectus deformities, broad thumbs, gastrointestinal defects such as anal anomaly with severe constipation, and a characteristic behavioral profile with friendly personality and short attention span (Clark et al., 2009; Opitz & Kaveggia, 1974). A recurrent mutation, p.R961W, in the MED12‐LS domain was identified in the original and five additional families with clinical features suggestive of FG syndrome (Risheg et al., 2007). Patients from the original Lujan syndrome family presented with X‐linked intellectual disability, hypotonia, macrocephaly, marfanoid habitus with long fingers with extensible joints, craniofacial features such as long and narrow face, high‐arched palate with hypernasal voice, and structural brain anomalies such as agenesis of corpus callosum (Lujan, Carlin, & Lubs, 1984). A single missense mutation in MED12‐LS domain, N1007S, has been identified in the original Lujan syndrome family (Schwartz et al., 2007). The patient with p.N898D mutation presented with features that mostly resemble FG syndrome; patients with p.R1214C and p.R1295H mutations presented with features that overlap with FG and Lujan syndromes; the patient with p.R206Q mutation showed severe psychiatric phenotype but without characteristic dysmorphic features or malformations of either FG syndrome or Lujan syndromes (Table 1). Taken together, our current studies on these four *MED12* mutations expand the genotype and phenotype spectrums of the *MED12*‐related X‐linked ID disorders.

MED12 consists of several structurally and functionally defined domains including a N‐terminal leucine‐rich domain (L), a leucine‐ and serine‐rich domain (LS), a proline‐, glutamine‐, and leucine‐rich domain (PQL), and a C‐terminal opposite paired domain (OPA). The PQL domain mediates protein–protein interaction while function for other domains remain largely unknown (Zhou et al., 2006). Classical FG and Lujan syndromes are caused by mutations in the LS domain, which is highly conserved (Figure 2a). Previous studies have shown that *MED12* mutations in FG and Lujan syndromes disrupt a mediator‐imposed constraint on GLI3‐dependent SHH signaling (Zhou et al., 2012). Expression levels of multiple SHH/GLI3 target genes including *GLI3, ASCL1, BMP4*, *CREB5*, and *NEUROG2*were significantly elevated in patients’ lymphoblast cell lines (Zhou et al., 2012). Increased expression of these genes was inhibited by a SHH antagonist, cyclopamine, suggesting that their induction was SHH/GLI3 dependent (Zhou et al., 2012).

By examining transcript levels of three Gli3‐dependent SHH‐signaling genes, *CREB5*, *BMP4*, and *NEUROG2,*in lymphoblasts, we found that p.N898D, p.R1214C, and p.R1295H mutations are associated with increased expression of all three genes. Interestingly, p.N898D mutation was found to associate with a significant increase in expression of *BMP4* but moderate increase in expression of *NEUROG22.*In contrast, p.R1214C and p.R1295H mutations were found to associate with significant increase in *NEUROG2* but moderate increase in *BMP4*. p.R206Q was found to associate with a mild increase in the expression of *BMP4* only. GLI3‐dependent SHH‐signaling pathway plays a critical role in development of multiple organs including brain, neural tube, developing limbs, and the gut (Villavicencio, Walterhouse, & Lannaccone, 2000). We speculate that severe disturbance in SHH‐signaling genes likely play an important role in craniofacial anomalies and multiple organ malformations in these patients. We further speculate that differences in the expression profile of SHH‐signaling genes potentially contribute to variable expression of phenotypes in patients with *MED12*‐related XLID syndromes.

The Ohdo syndrome is defined by several mutations in LS and PQL domains (p.R1148H, p.S1165P, p.A1383T, p.H1729N). Core clinical features of patients include moderate to severe intellectual disability, autistic behaviors, craniofacial anomalies such as microcephaly, blepharophimosis, prominent and bulbous nose, ear anomalies, and moderate to severe hypotonia. Skeletal, gastrointestinal, and genital urinary anomalies that are common in the FG‐Lujan syndrome spectrum are relatively mild or absent in patients with Ohdo syndrome. The molecular mechanisms that underlie Ohdo syndrome have not been fully characterized. MED12 has also been found to play key roles in the regulation of REST‐dependent epigenetic silencing of neuronal gene expression (Ding et al., 2008) and immediate early gene expression (Donnio et al., 2017). Systematic functional characterizations of these mutations are warranted.

## CONFLICT OF INTEREST

All authors declare no conflict of interest in the study.

## References

[mgg3569-bib-0001] Bouazzi, H. , Lesca, G. , Trujillo, C. , Alwasiyah, M. , & Munnich, A. (2015). Nonsyndromic X‐linked intellectual deficiency in three brothers with a novel MED12 missense mutation [c.5922G>T (p.Glu1974His)]. Clinical Case Reports, 3, 604–609. 26273451 10.1002/ccr3.301 PMC4527805

[mgg3569-bib-0002] Callier, P. , Aral, B. , Hanna, N. , Lambert, S. , Dindy, H. , Ragon, C. … Faivre, L. . (2013). Systematic molecular and cytogenetic screening of 100 patients with marfanoid syndromes and intellectual disability. Clinical Genetics, 84, 507–521. 10.1111/cge.12094 23506379

[mgg3569-bib-0003] Charzewska, A. , Maiwald, R. , Kahrizi, K. , Oehl‐Jaschkowitz, B. , Dufke, A. , Lemke, J. , … Kalscheuer, V. (2018). The power of the Mediator complex‐Expanding the genetic architecture and phenotypic spectrum of MED12‐related disorders. Clinical Genetics, 94, 450–456. 30006928 10.1111/cge.13412

[mgg3569-bib-0004] Clark, R. , Graham, J. J. , Friez, M. , Hoo, J. , Jones, K. , McKeown, C. , … Stevenson, R. (2009). FG syndrome, an X‐linked multiple congenital anomaly syndrome: The clinical phenotype and an algorithm for diagnostic testing. Genetics in Medicine, 11, 769–775. 10.1097/GIM.0b013e3181bd3d90 19938245 PMC4113033

[mgg3569-bib-0005] Ding, N. , Zhou, H. , Esteve, P.‐O. , Chin, H. , Kim, S. , Xu, X. , … Boyer, T. (2008). Mediator Links Epigenetic Silencing of Neuronal Gene Expression with X‐Linked Mental Retardation. Molecular Cell, 31, 347–359. 10.1016/j.molcel.2008.05.023 18691967 PMC2583939

[mgg3569-bib-0006] Donnio, L. , Bidon, B. , Hashimoto, S. , May, M. , Epantchintsev, A. , Ryan, C. , … Egly, J. (2017). *MED12*‐related XLID disorders are dose‐dependent of immediate early genes (IEGs) expression. Human Molecular Genetics, 26(11), 2062–2075. 10.1093/hmg/ddx099 28369444

[mgg3569-bib-0007] Graham, J. , & Schwartz, C. (2013). *MED12* related disorders. American Journal of Medical Genetics, 161A, 2734–2740. 24123922 10.1002/ajmg.a.36183 PMC3839301

[mgg3569-bib-0008] Ho, K. , & Scott, M. (2002). Sonic hedgehog in the nervous system: Functions, modifications and mechanisms. Current Opinion in Neurobiology, 12, 57–63. 10.1016/S0959-4388(02)00290-8 11861165

[mgg3569-bib-0009] Isidor, B. , Lefebvre, T. , Le Vaillant, C. , Caillaud, G. , Faivre, L. , Jossic, F. , … David, A. (2013). Blepharophimosis, Short Humeri, Developmental Delay and Hirschsprung Disease: Expanding the Phenotypic Spectrum of MED12 Mutations. American Journal of Medical Genetics, 164A, 1821–1825. 10.1002/ajmg.a.36539 24715367

[mgg3569-bib-0010] Kämpjärvi, K. , Kim, N. , Keskitalo, S. , Clark, A. , vonNandelstadh, P. , Turunen, M. , … Vahteristo, P. (2015) Somatic MED12 mutations in prostate cancer and uterine leiomyomas promote tumorigenesis through distinct mechanisms. Prostate, 76(1):22–31. 26383637 10.1002/pros.23092

[mgg3569-bib-0011] Langley, K. , Brown, J. , Gerber, R. , Fox, J. , Friez, M. , Lyons, M. , & Schrier Vergano, S. (2015). Beyond Ohdo Syndrome: A Familial Missense Mutation Broadens the MED12 Spectrum. American Journal of Medical Genetics, 167A, 3180–3185. 26338144 10.1002/ajmg.a.37354

[mgg3569-bib-0012] Lesca, G. , Moizard, M.‐P. , Bussy, G. , Boggio, D. , Hu, H. , Haas, S. , … Lespinasse, J. (2013). Clinical and Neurocognitive Characterization of a Family With a Novel MED12 Gene Frameshift Mutation. American Journal of Medical Genetics, 161A, 3063–3071. 24039113 10.1002/ajmg.a.36162

[mgg3569-bib-0013] Lujan, J. , Carlin, M. , & Lubs, H. (1984). A form of X‐linked mental retardation with marfanoid habitus. American Journal of Medical Genetics, 17, 311–322. 10.1002/ajmg.1320170124 6711603

[mgg3569-bib-0014] Niranjan, T. , Skinner, C. , May, M. , Turner, T. , Rose, R. , Stevenson, R. , … Wang, T. (2015). Affected Kindred Analysis of Human X Chromosome Exomes to Identify Novel X‐Linked Intellectual Disability Genes. PLoS ONE, 10, e0116454 10.1371/journal.pone.0116454 25679214 PMC4332666

[mgg3569-bib-0015] Opitz, J. , & Kaveggia, E. (1974). Studies of malformation syndromes of man 33: The FG syndrome. An X‐linked recessive syndrome of multiple congenital anomalies and mental retardation. Zeitschrift Für Kinderheilkunde, 117, 1–18. 4365204 10.1007/BF00439020

[mgg3569-bib-0016] Ravegnini, G. , Mariño‐Enriquez, A. , Slater, J. , Eilers, G. , Wang, Y. , Zhu, M. , … Fletcher, J. (2013). MED12 mutations in leiomyosarcoma and extrauterine leiomyoma. Modern Pathol, 26, 743–749. 10.1038/modpathol.2012.203 23222489

[mgg3569-bib-0017] Raymond, F. , Tarpey, P. , Edkins, S. , Tofts, C. , O'Meara, S. , Teague, J. , … Barthorpe, s., Buck, G. and al, e., (2007). Mutations in ZDHHC9, which encodes a palmitoyltransferase of NRAS and HRAS, cause X‐linked mental retardation associated with a marfanoid habitus. American Journal of Human Genetics, 80, 982–987. 10.1086/513609 17436253 PMC1852737

[mgg3569-bib-0018] Risheg, H. , Graham, J. J. , Clark, R. , Rogers, R. , Opitz, J. , Moeschler, J. , … Friez, M. (2007). A recurrent mutation in MED12 leading to R961W causes Opitz‐Kaveggia syndrome. Nature Genetics, 39, 451–453. 10.1038/ng1992 17334363

[mgg3569-bib-0019] Rocha, P. , Scholze, M. , Bleiss, W. , & Schrewe, H. (2010). Med12 is essential for early mouse development and for canonical Wnt and Wnt/PCP signaling. Development, 137, 2723–2731. 10.1242/dev.053660 20630950

[mgg3569-bib-0020] Schwartz, C. , Tarpey, P. , Lubs, H. , Verloes, A. , May, M. , Risheg, H. … Stevenson, R. (2007). The original Lujan syndrome family has a novel missense mutation (p. N1007S) in the MED12 gene. Journal of Medical Genetics, 44, 472–477. 10.1136/jmg.2006.048637 17369503 PMC2597996

[mgg3569-bib-0021] Shin, C. , Chung, W. , Hong, S. , Ober, E. , Verkade, H. , Field, H. , … Stainier, D. (2008). Multiple roles for *Med12* in vertebrate endoderm development. Developmental Biology, 317, 467–479. 10.1016/j.ydbio.2008.02.031 18394596 PMC2435012

[mgg3569-bib-0022] Tarpey, P. , Smith, R. , Pleasance, E. , Whibley, A. , Edkins, S. , Hardy, C. , … Stratton, M. R. . (2009). A systematic, large scale resequencing screen of X‐chromosome coding exons in mental retardation. Nature Genetics, 41, 535–543. 10.1038/ng.367 19377476 PMC2872007

[mgg3569-bib-0023] Tzschach, A. , Grasshoff, U. , Beck‐Woedl, S. , Dufke, C. , Bauer, C. , Kehrer, M. , … Bauer , P. . (2015). Next‐generation sequencing in X‐linked intellectual disability. European Journal of Human Genetics, 23, 1513–1518. 10.1038/ejhg.2015.5 25649377 PMC4613482

[mgg3569-bib-0024] Villavicencio, E. , Walterhouse, D. , & Lannaccone, P. (2000). The Sonic Hedgehog–Patched–Gli Pathway in Human Development and Disease. American Journal of Human Genetics, 67, 1047–1054. 10.1016/S0002-9297(07)62934-6 11001584 PMC1288546

[mgg3569-bib-0025] Vulto‐van Silfhout, A. , de Vries, B. , van Bon, B. , Hoischen, A. , Ruiterkamp‐Versteeg, M. , Gilissen, C. , … de Brouwer, A. (2013). Mutations in MED12 Cause X‐Linked Ohdo Syndrome. American Journal of Human Genetics, 92, 401–406. 10.1016/j.ajhg.2013.01.007 23395478 PMC3591845

[mgg3569-bib-0026] Wang, X. , Yang, N. , Uno, E. , Roeder, R. , & Guo, S. (2006). A subunit of the mediator complex regulates vertebrate neuronal development. Proceedings of the National Academy of Sciences, 103, 17284–17289. 10.1073/pnas.0605414103 PMC1859923 17088561

[mgg3569-bib-0027] Yamamoto, T. , & Shimojima, S. (2015). A novel MED12 mutation associated with non‐specific X‐linked intellectual disability. Human Genome Variation, 2, 15018 10.1038/hgv.2015.18 27081531 PMC4785543

[mgg3569-bib-0028] Yin, J. , & Wang, G. (2014). The Mediator complex: A master coordinator of transcription and cell lineage development. Development, 141, 977–987. 10.1242/dev.098392 24550107

[mgg3569-bib-0029] Zhou, H. , Kim, S. , Ishii, S. , & Boyer, T. (2006). Mediator modulates Gli3‐dependent Sonic hedgehog signaling. Molecular and Cellular Biology, 26, 8667–8682. 10.1128/MCB.00443-06 17000779 PMC1636813

[mgg3569-bib-0030] Zhou, H. , Spaeth, J. , Kimb, N. , Xu, X. , Friezc, M. , Schwartz, C. , & Boyerb, T. (2012). *MED12* mutations link intellectual disability syndromes with dysregulated GLI3‐dependent Sonic Hedgehog signaling. Proceedings of the National Academy of Sciences, 109, 19763–19768. 10.1073/pnas.1121120109 PMC3511715 23091001

