# Supplementary figures and images for: Dysregulations of sonic hedgehog signaling in MED12‐related X‐linked intellectual disability disorders
Source: Mol Genet Genomic Med. 2019 Feb 6;7(4):e00569. doi: 10.1002/mgg3.569 (PMC6465656; doi:10.1002/mgg3.569)

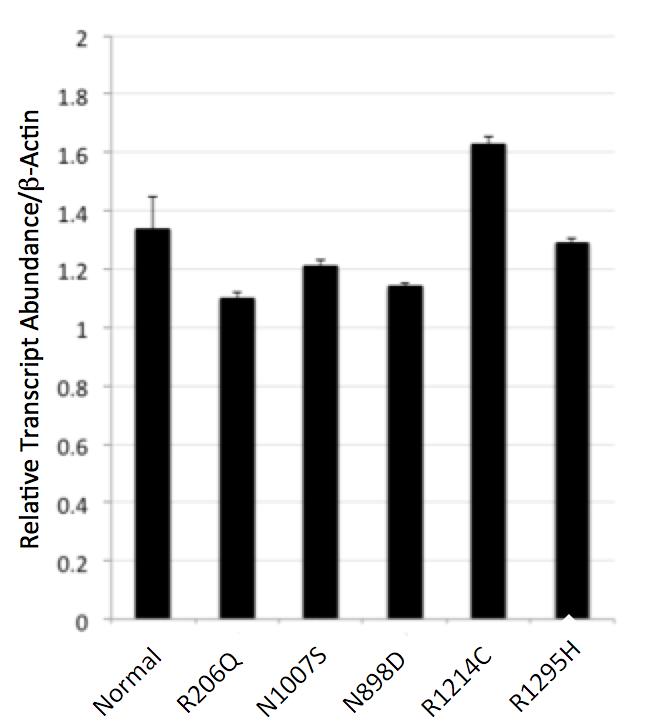


Srivastava et al., Figure S1

Supplement: Supplementary file 1 [file MGG3-7-na-s001.docx]
