# Supplementary material for: Dysregulations of sonic hedgehog signaling in MED12‐related X‐linked intellectual disability disorders
Source: Mol Genet Genomic Med. 2019 Feb 6;7(4):e00569. doi: 10.1002/mgg3.569 (PMC6465656; doi:10.1002/mgg3.569)
Supplement: Supplementary file 2 [file MGG3-7-na-s002.docx]

|  |  |  |
| --- | --- | --- |
| **Gene** | **Forward Primer (5’-3’)** | **Reverse Primer (5’-3’)** |
| **β-ACTIN (*ACTB*)** | 5’-AATGTGGCCGAGGACTTTGATTGC-3’ | 5’-AGGATGGCAAGGGACTTCCTGTAA-3’ |
| ***BMP4*** | 5’-TGCAGACCCTGGTCAATTCTGTCA-3’ | 5’-AGCATGGAGATGGCACTCAGTTCA-3’ |
| ***CREB5*** | 5’-CGTGCCTCCTTGAAACAAGCCATT-3’ | 5’-ATGAAACACCAGCACCTGCCTAGA-3’ |
| ***NGN2*** | 5’-AGGGCAGGTGTAGCCTTTCTGATT-3’ | 5’-CGCCACCCTTGGCTTTGACAATAA-3’ |
| ***HPRT1*** | 5’-CAAAGATGGTCAAGGTCGCAAGC-3’ | 5’-CCAGATGTTTCCAAACTCAACTTGAA-3’ |
|  |  |  |
|  |  |  |
|  |  |  |
|  |  |  |

Srivastava et al., Table S1
